# Perceptions of HIV and Mental Illness as “Western” or “Traditional” Illnesses: A Cross-Sectional Study from Limpopo Province, South Africa

**DOI:** 10.21203/rs.3.rs-3068420/v1

**Published:** 2023-06-26

**Authors:** Michael Galvin, Lezanie Coetzee, Patricia Leshabana, Nthabiseng Masebe, Shitshembiso Lebepe, Aneesa Moolla, Amanda R. Tarullo, Peter C. Rockers, Denise Evans

**Affiliations:** University of the Witwatersrand; University of the Witwatersrand; University of the Witwatersrand; University of the Witwatersrand; University of the Witwatersrand; University of the Witwatersrand; Boston University; Boston University; University of the Witwatersrand

## Abstract

Although Western biomedical treatment has dramatically increased across sub-Saharan African health systems, traditional medicine as a form of healing and beliefs in supernatural powers as explanations for disease remain prevalent. Research in this region has identified HIV in particular as a disease located within both the traditional African and Western medical paradigms, whilst mental illness is ascribed to primarily supernatural causes. Within this context, this study sought to understand and explore the perceptions of HIV and mental illness among a population of rural women in Limpopo, South Africa. 82 in-depth interviews were conducted between January and December, 2022. Interviews were transcribed and translated into English. Data were managed using NVivo 11 software and thematically analyzed. The majority of participants identified HIV as a Western illness requiring biomedical treatment with causation largely attributed to biological mechanisms. A traditional form of HIV only cured using traditional treatments was also denoted. Unlike for HIV, the majority of respondents felt that there was no biological or behavioral cause for mental illness but rather the illness was conceptualized supernaturally thus likely impacting patient care pathways. Further research to study HIV and mental health perceptions among a larger sample in different regions of sub-Saharan Africa is warranted.

## INTRODUCTION

Many scholars are currently examining the subject of health beliefs and their implications for access to healthcare and disease treatment in sub-Saharan Africa (Thornton, 2017; Sundararajan et al., 2020; Ayinde et al. 2021). Although Western biomedical explanations and treatment for illness and disease have slowly been incorporated into African health systems over the last centuries, beliefs in supernatural powers as explanations for illness and misfortune remain prevalent (van der Zeijst et al., 2021). Researchers have long assessed illness in the sub-Saharan context through the lens of a dichotomization between so-called “Western” versus “traditional” ailments (Haram, 1991; Steen & Mazonde, 1999; Okello & Neema, 2007; Kovačič, 2020; Pemunta & Tabenyang, 2020).

Many studies have examined the perceived supernatural etiology of illness in the sub-Saharan context, highlighting how conditions such as mental illness and other non-communicable diseases have been commonly ascribed to spirit possession (Carrazana et al., 1999; Ovuga et al., 1999; Ngoma et al., 2003; Sorsdahl et al., 2009). Yet, outside of anthropology research, these alternative explanatory models only began to receive significant attention with the emergence of HIV/AIDS in Africa, particularly starting in the mid-1980s (Van Dyk, 2001; Tenkorang et al., 2011). This was a period characterized by fear, denial, and confusion as to the cause of HIV/AIDS, as large numbers of otherwise young and healthy people were inexplicably wasting away and dying (Ashforth, 2005).

Studies in South Africa highlighted how AIDS was often interpreted in line with other “spiritual ailments” – such as mental illness – as a “discourse of witchcraft” among a majority of South Africans (Van Dyk, 2001; Delius & Glaser, 2005; Rödlach, 2006). Others describe a “dual belief system” in which a “traditional” belief-based explanatory model of disease was held in parallel to a “Western” germ theory model of HIV, often within the same individual (Pascoe et al., 2013). However, this same study maintains that beliefs around the cause and origin of HIV were more often located in the “traditional” as opposed to the “Western” realm, highlighting the extent to which many South Africans continued to blame bewitchment and other traditional explanations for the large numbers of people dying of AIDS in their communities.

Nevertheless, as antiretroviral therapy (ART) began to become more widely available in many African countries throughout the 2000s and 2010s due to large efforts from the international community, deaths rates decreased significantly and more patients began to live with HIV (Varmus, 2013). Many describe the heavily mediatized phenomenon of the “Lazarus effect” during this period – a biblical allusion to the raising of Lazarus from the dead – in which AIDS patients who took ART “miraculously” recovered from near death, regained normal weight, and resumed a normal life (Beyrer et al., 2009; Compassion, 2012; Rasmussen & Richey, 2012). Despite these advances, few studies have examined the transformation in the perception of HIV among populations in sub-Saharan Africa. Additionally, while significant medical developments have changed the treatment of HIV in recent decades, scholars continue to note that other non-communicable illnesses, such as mental illness, continue to be considered “traditional” in origin by a vast majority of populations throughout sub-Saharan Africa (Ayinde et al., 2021; Ojagbemi & Gureje, 2021).

“Traditional” versus “Western” explanatory models for illness represent an important question in sub-Saharan Africa for several reasons. Firstly, several studies have shown that pathways patients follow when seeking healthcare for mental illness can cause delays in successful treatment (Mkize, 2001; Ibrahim et al., 2016; Drury, 2020). In this sense, traditional African sociocultural beliefs which perceive illness as arising from supernatural factors such as offenses against God, ancestors, or bewitchment, can lead individuals to discount the effectiveness of evidence-based interventions (Adeosun et al., 2013). One study in South Africa found that spiritual attribution of cause and consultation with traditional healers was associated with a longer duration of negative psychiatric symptoms (Burns et al., 2011). Thus, it is important to consider explanatory models and their implications when developing awareness raising, community engagement, or public education campaigns around healthcare access and care seeking behaviors in this region.

This study builds on previous research by examining the perceptions of illnesses such as HIV and mental illness in the context of religio-cultural beliefs and practices in sub-Saharan Africa, and South Africa in particular. As this study targeted women in a rural area of Limpopo province, this research sought to examine the perspectives of more marginalized populations who are among the most in need of additional health education campaigns and services. This research thus aimed to examine several questions: (1) To what extent does HIV continue to be viewed as a traditional illness despite significant advances in treatment and care in recent decades? (2) To what extent does mental illness continue to be viewed as a traditional illness? (3) How does HIV compare with illnesses such as mental illness, which have not had significant advances in treatment during this period? (4) What role does location, gender, and cultural background play in these perceptions? (5) And lastly, what implications do these findings have for future public health campaigns?

## METHODS

This was a cross-sectional qualitative study, nested in a larger study called The Developing Belief Network (DBN) Study which is conducting research at sites across the globe. The data collected for this particular study was nested in the research conducted in Tzaneen, Limpopo province, South Africa, in 2022. The goal of the overall DBN study is to document cultural influence on children’s (a) attainment and construction of religious knowledge or behaviors, and (b) conveyance of this religious knowledge and these practices to other individuals. Other questions the DBN study sought to answer include (1) How does the acquisition of religious cognition and behaviour vary within and between populations? and (2) How do processes of social learning support the development of religious cognition and behaviour?

This study utilized a snowball sampling strategy in which community health workers (CHWs) at six local primary health care (PHC) facilities that were participating in the study first worked with the study team to identify potential participants. Members of the study team asked participants if they knew of anyone else who would be interested in taking part in the study. If yes, the participants provided contact details in order to contact new potential study participants via telephone for recruitment. All communication and study documents were conducted in local languages (i.e., English, Xitsonga, and Sepedi).

Between June 22, 2022 and December 8, 2022, participants who were willing to participate in the study spoke with a member of the study team who determined if they were eligible to participate. Participants were included in this study if they were: 1) caregivers of children between 4–10 years of age; 2) 18 years or older; 3) and fluent in Xitsonga, Sepedi, or English. It is important to not that these eligibility criteria were for the larger Developing Belief Network study and caregivers were interviewed for these current analyses as a convenience sample. Interviews were all conducted at study offices and patients were provided transportation to and from their homes. Participants were provided a refreshment and a snack during the visit as well.

Those participants deemed eligible for the study were administered informed consent. They were presented with the information sheet and it was explained to them in detail by the study staff, after which they had the opportunity to ask questions. Spaces were established in study offices in which to consent participants and conduct the interviews privately. Participants understood that they were free to withdraw from the study at any time. Participants who were willing to continue with the interview were asked to sign the study consent form as well as an audio consent form. Individual once-off interviews took roughly 45 minutes to complete. Participants received a small reimbursement (R150 or equivalent to 10 United States Dollars) to compensate them for their time and any out-of-pocket expenses they may have incurred.

Participants were asked about their age, monthly household income, employment status, education level, ethnic group, religious affiliation, marital status, and number of children they have. Participants were also asked questions such as “Do you believe mental illness is a Western illness, a traditional illness, or both?” and “Why?” Additionally, they were asked “Do you believe HIV is a Western illness, a traditional illness, or both?” and “Why?” The words “Xilungu” and “Sekgowa” were used in Xitsonga and Sepedi respectively, and refer to “white people’s customs,” or “Western/English” culture. The words “Xintu” and “Ndhavuko” were used in Xitsonga, and refer to “traditional” or “cultural” customs, and the words “Dilo tsa sesotho” were used in Sepedi, referring to “traditional things/practices.”

All interviews were audio recorded and transcribed. All participants consented to be audio recorded. Three study enumerators reviewed all transcripts and translated them into English. The analysis utilized a directed content analysis method that combined deductive and inductive aspects of code development (Hsieh & Shannon, 2005). Themes were developed and refined by the three study enumerators and study PI resulting in the generation of a final set of codes. Researchers subsequently utilized NVivo 11.0 to code the transcripts. The survey data was collected and stored in REDCap, an electronic data capture tool hosted by the University of the Witwatersrand, so as to maintain confidentiality.

The study was approved by the Human Ethics Research Committee (Medical) at the University of the Witwatersrand, Johannesburg (Clearance number: M210914).

## RESULTS

This study examined a population of rural women living nearby the city of Tzaneen. Eighty-two (n = 82) individuals completed the study interview and were therefore included for this analysis.

This study explored the perceptions of HIV and mental illness among a population of rural women in northern South Africa’s Limpopo province. Participants ranged between the ages of 18 and 60 years of age (Table 1). The population of this study was largely poor with 88% making a household income of less than 5,000 ZAR (roughly $275) per month, and only 20% were employed. However, as these are caregivers of young children, it is perhaps not surprising that many of them were not formally employed. Over 60% of the sample had a primary education or less. The sample was relatively evenly split between Tsonga (part of the Bantu ethnic group) and Pedi ethnic groups. Eighty-seven percent (87%) identified as Christian, while 9% identified as traditional, and 4% as other.

**Figure 1.**
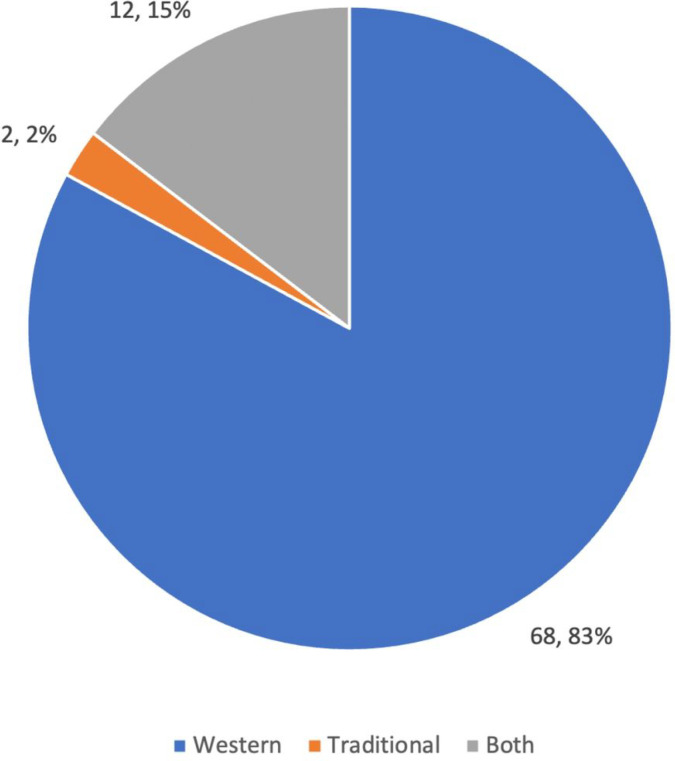
Is HIV a Western or traditional illness?

**Figure 2.**
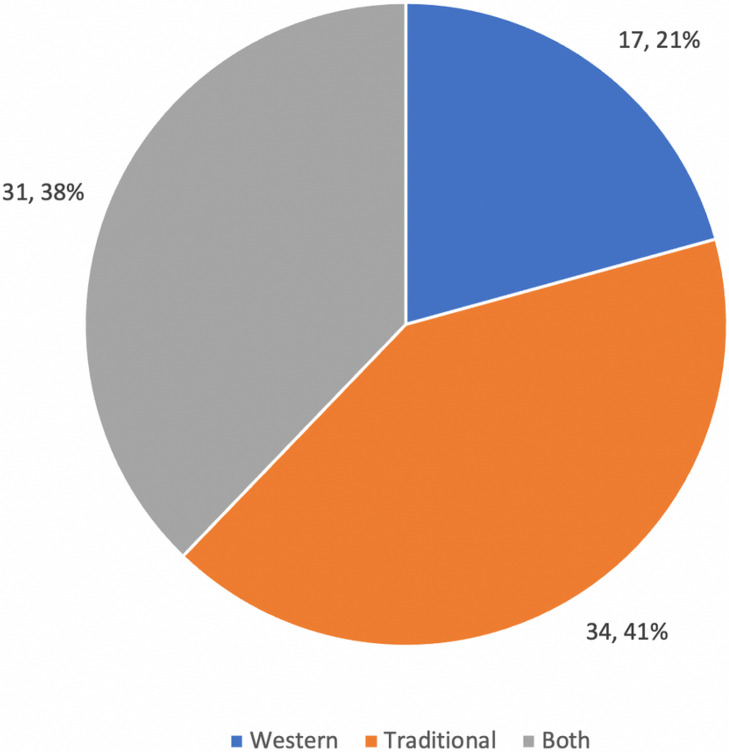
Is mental illness a Western or traditional illness?

While only 2% of participants identified HIV as a traditional illness, 42% identified mental illness as a traditional illness. Similarly, whereas 83% said HIV was a Western illness, only 21% said mental illness was a Western illness. Lastly, 38% of participants said mental illness could be both Western or traditional, and 15% said the same for HIV. See Figs. 1 and 2. The questions “Is HIV a Western or traditional illness” and “Is mental illness a Western or traditional illness” did not differ by any other characteristics including education, employment, ethnic group, monthly income, religion, age, or number of children.

### HIV/AIDS

HIV/AIDS was overwhelmingly described as a Western illness as opposed to a traditional illness by study respondents. There were many ways in which participants expressed this view. “It comes in a Western way,” one said (Participant 3). “It isn’t traditional,” said another (P68). Or simply, “you won’t get HIV through witchcraft” (P7).

Rather, causation was overwhelmingly attributed to behavior, with respondents describing how “it’s caused by people who don’t use protection when having sex, or maybe if the condom bursts” (P35). Thus, most participants argued that patients with HIV could not get help from traditional healers but rather needed to go to the clinic for biomedical care: “I have never heard of anyone who had HIV and went to a traditional healer and got better, but if they go to the clinic they can get help” (P76).

Nevertheless, a small minority of participants still felt there could be traditional causation or treatment for HIV. “It’s natural, it’s not caused by people,” one participant remarked (P47). Similar to how respondents described mental illness due to traditional causes, they remarked that, while usually a Western illness, there are forms of HIV that can be traditional in origin. As one describes, “they [traditional healers] can infect you traditionally, for example they can put it through the door at [your] home” (P57). Another remarked, “they can take the blood of someone who is HIV positive and put it in the body of someone who is negative, spiritually” (P64). In this sense, traditional forms of HIV are conceptualized as being transmitted supernaturally.

Similar to its non-Western causation, the traditional form of HIV is also incompatible with Western treatment: “I can test positive at the hospital and take treatment [there], then when I go back for a checkup they tell me I’m testing negative because it [the HIV] was created by people [witchcraft]” (P37). Another supported this idea saying, “they can send you an illness through witchcraft, and when you go to the hospital they can’t find the illness. The patient becomes surprised because they are taking treatment [ART] but the immune system doesn’t respond” (P66). This spiritual form of HIV could only be cured spiritually, as one respondent noted that she saw a patient cured through prayer: “they prayed for him and he got better” (P22). Nevertheless, the number of participants viewing HIV as traditional were very low with over more than 4 out of 5 viewing it as Western only.

### Mental Health

The minority of participants describing mental illness as a Western illness attributed this to either stress, drugs, or biology. Firstly, stress was often referred to by using the idiom of distress “thinking too much,” “overthinking,” or “problems of thinking.” As one respondent described, “a person can lose their mind when they have too much stress, when they’re thinking too much to a point where people end up saying ‘you are crazy’” (P12). Stress was a commonly perceived reason for mental illness, with participants describing how stress can “disturb” people or “make them lose their minds.” Others referred to mental illness due to stress as something only “white people” get (P34). “If it is a white person it will be because of depression, but for us it’s a result of witchcraft” (P65).

Besides stress and thinking too much, others described mental illness as a Western illness that can result from drug use. Most often this was related to marijuana use, or “dagga” as it is referred to locally. After using dagga, respondents discussed how, for some people, “the brain just ends up losing it” resulting in mental illness (P33). On the other hand, environmental causes such as stress and drugs were not the only explanations for why mental illness could be a Western illness. Two respondents referred to biology as a cause, saying that “people are just born that way” sometimes (P7, P16).

Most participants reported that mental illness is more likely to be a traditional illness however, and due to bewitchment. Bewitchment was often referred to as illness that is “caused by people.” As one described, mental illness is “mostly caused by people, because some are born healthy then they suddenly go crazy” (P30). Others described bewitchment by saying “they made you that way,” referring to traditional healers. “Traditionally they just make you like that,” one remarked (P32).

Participants explained that mental illness can be caused traditionally either because the person “deserves it” – thus, as a punishment – or not – usually due to jealousy. At times, a person will become mentally ill as a way to punish them via traditional means. Several respondents noted how theft in particular, can be a reason for bewitchment. As one describes, “a person can grow up normal, but when he starts being naughty by stealing from people, they [traditional healers] will make him go crazy by bewitching him” (P40). Another referred to people doing “immoral things” often becoming mentally ill, also emphasizing bewitchment as a traditional form of punishment.

On the other hand, people can be bewitched without “deserving it.” As one respondent said, “it’s because someone was going to succeed in life and people didn’t want that” (P43). Another states, “witches are driven by jealousy and you find that I may become successful so they make me go crazy. It usually happens to those who are well educated. When they see that you are intelligent they mess with your brain and make you go crazy” (P1). This link between education, success, and jealousy was often cited. As one participant remarked, “I knew someone who was intelligent and then went crazy; they made him crazy because they saw his future and had to ruin it for him” (P9).

## DISCUSSION

This study found that the vast majority of the women interviewed in rural Limpopo province, South Africa identified HIV as a Western illness which necessitates treatment from biomedical healthcare providers, such as those in hospitals and clinics. Causation was perceived to be overwhelmingly through biological mechanisms such as through sexual activity in which protection either is not used or fails – for example, when a condom breaks during sexual intercourse. While a small minority of participants identified HIV as possibly being of traditional or “supernatural” origins – such as bewitchment – this was usually through describing HIV as having a sort of dual identity. Other research has identified this belief in “doppelganger illnesses” where traditional illnesses simply resemble Western diseases (Pascoe et al., 2013; Feldman et al., 2021; Nicolini, 2022). In this sense, HIV is considered to be a Western illness, but there are also traditional illnesses that mimic its symptomology, thereby meriting the term doppelganger.

As opposed to HIV, among participants of this study, mental illness continues to be conceptualized as a largely traditional ailment in which bewitchment is identified as the predominant etiology. Reflecting this, a large majority of participants in this study described mental illness as arising from supernatural forces in which the illness is sent onto someone else via witchcraft. While occasionally this was described as a form of punishment for individual wrongdoing, in many cases the illness was simply as a result of jealousy that the perpetrator had for the victim. Parallels can be made with studies identifying the phenomenon of “crab antics” which has been described primarily in Caribbean research (Punski-Hoogervorst et al., 2021). Thus, unlike HIV, respondents felt that there was less of a biological or behavioral cause for mental illness – such as a condom breaking during sex – but rather the illness was more likely to be conceptualized supernaturally.

Many other studies have examined in depth the way religio-cultural concepts of bewitchment interact with forms of illness and misfortune in this context (Kabamba, 2014; Thornton, 2017; Mayston et al., 2020; Netshapapame et al., 2021; Ojagbemi & Gureje, 2021). Yet, very little research currently exists on the relationship between cultural beliefs and the transformation of explanatory models of illnesses such as HIV over time in sub-Saharan Africa. One recent study from Botswana argues however that traditional explanations for HIV and tuberculosis may have moved away from witchcraft in recent years due to the increased scope of biomedical treatments available (Becker et al., 2019). This same study also notes that ailments such as mental illness remain strongly related to traditional beliefs in part because of the limited range of biomedical treatments. These findings may therefore reflect a dichotomy between communicable and non-communicable illnesses, of which the latter are generally less likely to have pharmacological treatments.

Another study in South Africa examined changing perceptions surrounding HIV disclosure during the past decade, finding that – as HIV shifts from a “death sentence” to a “chronic condition” – many find fewer negative consequences of disclosing their status (Schatz et al., 2022). Though this study did not examine perceptions of traditional beliefs and HIV, it highlights other social changes that have resulted due to the impacts of ART on longevity and HIV stigma. However, despite these populations-level changes in perceptions and beliefs surrounding HIV, many South African patients continue to seek treatment with traditional healers and others who practice outside the biomedical paradigm (Bosire et al., 2022). This often results in faster disease progression, poorer outcomes, and negative financial impacts.

Researchers currently argue that beliefs regarding the supernatural etiology of illness may predominate and persist when a specific disease is considered “difficult” to treat (Becker et al., 2019; Galvin & Michel, 2020). Other studies in the past have also found that when symptoms are deemed treatable by Western medicine – such as with leprosy – it is more likely to be sought out by local populations (Kumaresan & Maganu, 1994; Steen & Mazonde, 1999). While this may seem like a relatively self-evident observation, it has significant implications for disease treatment – both for illnesses like HIV and mental illness – throughout sub-Saharan Africa. In particular, it supports the notion that awareness raising, patient education, and sharing patient experiences can improve care-seeking behaviors. In addition, as this study targeted women in a rural area of Limpopo province, this research aimed to examine the perspectives of marginalized populations – women and people living in more isolated areas less connected to cities – who tend to be the most in need of health education campaigns and services.

This study has several limitations. First of all, as this study used snowball sampling and was qualitative and isolated to one area of Limpopo province and only interviewed women, it is not meant to be generalizable. For example, another study in South Africa from 2010 found discrepancies in belief between rural regions, with 85% of interviewees in one area believing “God can cure HIV” whereas only 40% did in another region of the country (Landman, 2020). Second, this study is cross-sectional and, therefore cannot determine causality or changes in HIV perceptions over time. Third, there may be some social desirability bias with patients saying what they believe staff wants to hear so as not to risk involvement in other waves of data collection or in receiving their reimbursement. However, it is not clear in which direction this would have impacted the data. Lastly, non-communicable illnesses such as mental illness were probably always more likely to be considered traditional than infectious diseases such as HIV/AIDS; however, the large disparity uncovered by this research indicates a strong likelihood that perceptions of HIV as a traditional illness have changed significantly in recent decades. More research is needed to fully assess the constantly evolving relationship between traditional beliefs and different illnesses in South Africa.

## CONCLUSION

This study aimed to examine the perceptions of a population of rural women in Limpopo province, South Africa regarding the etiology of HIV and mental illness. In particular, this research sought to understand the extent to which respondents considered both illnesses either “Traditional” or “Western” in origin. While the vast majority considered HIV a “Western illness” – in which those infected should only seek biomedical treatment – many believed the opposite of mental illness attributing it largely to supernatural causes. This may result in delays in seeking care and community stigma around mental illness. Additional education, awareness raising, and stigma reduction campaigns for mental illness could promote care seeking behavior and reduce community stigma.

With regards to HIV, while significant research from previous decades found similar explanatory models for HIV as mental illness, this may have shifted with the large-scale rollout of ART in sub-Saharan Africa over the past decade. Further research could study HIV perceptions among a larger sample in different regions of sub-Saharan Africa. Additionally, studies could examine changes over time in illness perception, particularly when significant biomedical developments occur – such as with the widespread availability of ART for HIV.

## Figures and Tables

**Table 1:** Sample Characteristics (n = 82)

	N (%)	Mean (SD)
Age (min/max: 18–61 years)	–	33 (11.4)
Monthly Income in ZAR		
Less than 5,000	72 (88%)	–
5,000 to 10,000	7 (8%)	–
10,000 or more	3 (4%)	–
Employment Status		
Employed	16 (20%)	–
Unemployed	63 (76%)	–
Retired	3 (4%)	–
Education		
No Formal Education	3 (4%)	–
Primary	47 (57%)	–
Secondary	27 (33%)	–
University	5 (6%)	–
Ethnic Group		
Tsonga	45 (56%)	–
Pedi	37 (44%)	–
Religion		
Christian	71 (87%)	–
Traditional	7 (9%)	–
Other	4 (4%)	–
Number of Children (min/max: 0–7)	–	3 (1.5)
Marital Status		
Single	50 (61%)	–
Married	25 (31%)	–
Divorced	6 (7%)	–
	**N (%)**	**Mean (SD)**
Widowed	1 (1%)	
